# Older caregivers’ responsibilities and strategies for their cohabiting partners living at home—a qualitative systematic literature review

**DOI:** 10.3389/fpubh.2025.1658095

**Published:** 2025-10-17

**Authors:** Stinne Glasdam, Hongxuan Xu, Ruth-Ellen Slåtsveen, Christie Stilwell, Gitte Wind, Ragnhild Julante Andersen Gulestø, Pier-Luc Turcotte

**Affiliations:** 1 Department of Health Sciences, Lund University, Lund, Sweden; 2 Department of Care Science, Malmo universitet, Malmö, Sweden; 3 Centre for Development of Institutional and Home Care Services in Oslo Kommune, Oslo, Norway; 4 Faculty of Health, Dalhousie University, Halifax, NS, Canada; 5 Department of Nursing and Nutrition, Kobenhavns Professionshojskole, Copenhagen, Denmark; 6 Department of Health Sciences, VID Vitenskapelige Hogskole, Oslo, Norway; 7 University of Ottawa, Ottawa, ON, Canada

**Keywords:** ageing-in-place, caregiver burden, cohabiting partners, older adults, qualitative systematic literature review, responsibilities, roles

## Abstract

**Background:**

Ageing-in-place policies have increasingly shifted elder care responsibilities onto family members. Among older cohabiting couples, one’s partner’s illness significantly impacts the other’s daily life and well-being.

**Aim:**

To explore the responsibilities and strategies of caregiving partners in older cohabiting couples from the perspectives of cohabiting caregivers.

**Methods:**

A qualitative systematic literature review was conducted across seven databases, following PRISMA guidelines and registered in PROSPERO (CRD42025632103). Sixty-five studies were included based on predefined inclusion and exclusion criteria using Covidence. Quality was assessed using the Critical Appraisal Skills Programme (CASP) checklist for qualitative studies. Data were synthesised through descriptive numerical summaries and thematic analysis.

**Results:**

The studies, conducted in 20 countries, primarily used individual interviews for data collection. Three overarching themes emerged: (1) strategies directed towards the partner, (2) strategies to maintain personal identity and space, and (3) strategies for navigating formal care systems. Cohabiting caregivers often assumed daily responsibilities despite emotional and physical strain. They relied on both informal and formal support to care for their partner and preserve time for themselves. However, formal care involvement led to issues such as broken agreements and inadequate services.

**Conclusion:**

Older cohabiting caregivers constantly balance and adapt their caregiving roles in relation to their partner, themselves, and formal care providers. This shift in responsibility results in an often invisible, morally-driven labour that remains under-recognised in Ageing-in-place policies. The study highlights the need for policy frameworks and interventions that acknowledge caregivers’ moral labour, enhance the quality of formal care, and support caregiver autonomy.

**Clinical trial registration:**

https://www.crd.york.ac.uk/PROSPERO/view/CRD42025632103, identifier (CRD42025632103).

## Introduction

Healthcare services are continuously evolving due to advancements in knowledge, changes in the population’s health conditions, and demographic shifts, creating a substantial and continuous influence on global population dynamics (1). As part of this evolving landscape, there is a shift away from institutional care as the conventional standard for older adults and contemporary trends indicate a growing preference for and acknowledgement of older adults’ preference for living at home into advanced age, also called ‘Ageing-in-place’ (1–3). In many western countries, governments have outlined Ageing-in-place policies that shift the responsibility of elder care from health professionals to family members (4–6). Ageing-in-place is generally presented as beneficial for the well-being and autonomy of older adults, given that remaining in familiar surroundings can improve quality of life and foster a sense of security and dignity (7–9). However, it can carry implicit expectations about life at home in old age (10, 11) that shape societal perceptions as to what a ‘good’ life means in old age (12, 13). Hence, concerns have arisen regarding the impact of discourses on successful, active, and healthy ageing on older adults (14), which often impose expectations on how older adults should lead their lives (11, 14) and overlook the fact that old age is not a homogeneous experience (15, 16).

Ageing-in-place policies also have complex implications, particularly as they increasingly rely on partners and other family members to provide necessary support (4, 17, 18, 126). Partners often serve as the primary source of care in later life (19, 20). The interaction between older couples and their environments influences how they experience and adjust to old age at home, creating a dynamic that is personal, social, and physical (21, 22). For instance, research highlights that couples often frame their frailty and health issues differently, depending on their collective outlook and shared memories, as they prepare for future possibilities, including end-of-life (23, 24). While many partners willingly assume caregiving responsibilities, it cannot be assumed that they are all enabled or willing to do so (25). In couples with low quality relationships, partners may not want to provide care (26). In addition, traditional gendered expectations significantly affect caregiving, where providing care for a partner is viewed as a feminine role for the wives rather than husbands (19, 27, 28). However, this is not always the case, as a study from the Netherlands found no gendered differences in the likelihood of older adults to receive care from their partner, rather it was influenced more by other factors such as the partner’s ability to provide care or relationship quality (29).

Frailty in one partner often leads to transformation in the partners’ relationship, where caring becomes integral to everyday life and is influenced by their unique life courses and histories together (30). Older couples frequently adapt to challenges through shared routines and mutual support, relying on long-standing companionship to help maintain each other’s wellbeing (27). Rather than restricting fulfilment, this shift in roles can deepen connection and purpose between partners, challenging assumptions about loss of independence (30–32). Partner caregiving often entails a dual role, as caregivers must also manage their own age-related health challenges (19, 33). This underscores the diverse ageing experiences, with the past and future continuing to shape partners’ care roles (23, 34). While caregiving can be deeply meaningful, it presents distinctive physical, emotional, and social strains, especially as both partners experience the ageing process individually and together. Many older adults depend on their partners for support, whether by choice or financial necessity, creating a unique caregiving dynamic where both partners may confront similar physical or cognitive limitations (22, 27, 28), though to varying extents as one is able to fully or partially care for the other at home.

Recent literature reviews characterise informal caregiving for frail or ill older adults, such as those living with dementia, multimorbidity, or undergoing cancer treatment, as a demanding and often burdensome job (18, 27, 35–39). Caregivers manage daily activities, face a gradual erosion of reciprocity in their relationships, and risk social isolation. At the same time, they are often required to provide instrumental, preventive, and emotional care, frequently at the expense of their own well-being and personal routines. While some studies highlight positive aspects, such as personal growth, enhanced relational closeness, and family cohesion, the literature remains fragmented, particularly regarding how cohabiting partner caregivers understand and manage their responsibilities and sustain caregiving in everyday life. This reveals a significant knowledge gap concerning the everyday practices and strategies of cohabiting caregivers navigating these complex roles. Through a review of the existing literature, this study aims to explore the responsibilities and strategies of the caregiving partners in older couples living at home, from the perspectives of cohabiting older adults.

## Method

This study carried out a qualitative systematic review to synthesise insights from qualitative studies, following a method adapted from Bettany-Saltikov and McSherry (40). It is conducted in line with the Preferred Reporting Items for Systematic Reviews and Meta-Analyses (PRISMA) guidelines (41), and the review protocol is registered with PROSPERO (registration number CRD42025632103).

### Identifying the research questions

This review aimed to answer the following three research questions:

(1) What are the well-being and health conditions of cohabiting older adults living at home?
(2) What responsibilities did cohabiting caregivers have for their partner and themselves in daily life?
(3) What strategies did cohabiting caregivers use to make daily life work for themselves and their partner?

### Inclusion and exclusion criteria

The inclusion criteria were: (1) Studies about responsibilities and strategies of the caregiving partners in older couples living at home, (2) Perspectives of older cohabiting caregivers and care receivers at home, age 60 + years old, (3) Qualitative studies or qualitative sub-studies in mixed method studies, (4) Published in English, French, or Scandinavian languages, and (5) Published between 1 January 2015–28 January 2025 to align with the latest evidence related to the study’s aim. The review excluded: (1) Systematic literature reviews, (2) Intervention studies, (3) Editorials/commentaries, (4) Dissertations/theses, and (5) Guidelines/recommendations. Old age is defined differently across academic traditions and countries, with thresholds ranging from 50 to 70 years. For this study, we included studies involving adults aged 60 and older, as our preliminary literature search identified this age range as the most used definition.

### Searching, selecting, appraising, and extracting relevant data

A search was performed in the PubMed, EMBASE, CINAHL Complete, Eric, SocINDEX, and PsycInfo databases with support from an experienced librarian (Last search: 28 January 2025). Inclusion and exclusion criteria were defined according to the Population, Exposure, and Outcome (PEO) model. The PEO model was selected because it offers a structured approach to framing research questions and organising data, which aligns effectively with qualitative methodologies (40, 42). The search strategy followed the building block approach structured around the PEO model (Table 1).

**Table 1:** Populations, exposures, and outcomes (PEO).

Population (P)	Exposure (E)	Outcome/Theme (O)
Older caregivers caring for their partners	Home care environment in primary care	Responsibilities and strategies of the caregiving partners from the perspectives of cohabitant older caregivers and partners

Search terms within each block were tailored to suit the specific requirements of each database. Details of the search strategies are provided in Table 2.

**Table 2:** Search strategies.

Search line #	Search terms	Results
PubMed		
#1	Aged[Mesh]OR aged[Title/Abstract] OR old[Title/Abstract] OR older[Title/Abstract] OR elder[Title/Abstract] OR older adults[Title/Abstract] OR senior[Title/Abstract] OR seniors[Title/Abstract] OR octogenarian*[Title/Abstract] OR pensioner*[Title/Abstract] OR dementia*[Title/Abstract]	5,634,835
#2	caregiv*[Title/Abstract] OR care giv*[Title/Abstract] OR care provid*[Title/Abstract] OR care staff[Title/Abstract] OR community care[Title/Abstract] OR partner*[Title/Abstract] OR spouse*[Title/Abstract] OR cohabit*[Title/Abstract] OR "co-habit*"[Title/Abstract] OR relative*[Title/Abstract] OR famil*[Title/Abstract] OR sibling*[Title/Abstract] OR sister*[Title/Abstract] OR brother*[Title/Abstract] OR general practitioner*[Title/Abstract] OR GP[Title/Abstract] OR GPs[Title/Abstract] OR occupational therapist*[Title/Abstract] OR physiotherapist*[Title/Abstract] OR informal carer*[Title/Abstract] OR couple*[Title/Abstract]	3,996,244
#3	"Home Nursing"[Mesh]OR home care[Title/Abstract] OR home nursing[Title/Abstract] OR care home*[Title/Abstract] OR ordinary hous*[Title/Abstract] OR ordinary accommodation*[Title/Abstract] OR living at home[Title/Abstract] OR Ageing in place[Title/Abstract] OR togetherness[Title/Abstract] OR relational turbulence[Title/Abstract]	42,645
#4	#1 AND #2 AND #3	10,280
#5	Filters: Danish, English, Norwegian, Swedish, from 2015 to 2025	4,337
Embase		
#1	'aged'/exp OR aged:ti,ab,kw OR 'old age':ti,ab,kw OR 'old adult*':ti,ab,kw OR 'old people':ti,ab,kw OR older:ti,ab,kw OR elder:ti,ab,kw OR older adults:ti,ab,kw OR senior:ti,ab,kw OR seniors:ti,ab,kw OR octogenarian*:ti,ab,kw OR pensioner*:ti,ab,kw OR dementia*:ti,ab,kw	5,547,282
#2	'caregiver'/exp OR caregiv*:ti,ab,kw OR 'care giv*':ti,ab,kw OR 'care provid*':ti,ab,kw OR 'care staff':ti,ab,kw OR 'community care':ti,ab,kw OR partner*:ti,ab,kw OR spouse*:ti,ab,kw OR cohabit*:ti,ab,kw OR 'co-habit*':ti,ab,kw OR relative*:ti,ab,kw OR famil*:ti,ab,kw OR sibling*:ti,ab,kw OR sister*:ti,ab,kw OR brother*:ti,ab,kw OR 'general practitioner*':ti,ab,kw OR gp:ti,ab,kw OR gps:ti,ab,kw OR 'occupational therapist*':ti,ab,kw OR physiotherapist*:ti,ab,kw OR 'informal carer*':ti,ab,kw OR couple*:ti,ab,kw	4,993,175
#3	'home care'/exp OR 'independent living'/exp OR 'home care':ti,ab,kw OR 'home nursing':ti,ab,kw OR 'care home*':ti,ab,kw OR 'ordinary hous*':ti,ab,kw OR 'ordinary accommodation*':ti,ab,kw OR 'living at home':ti,ab,kw OR 'ageing in place':ti,ab,kw OR togetherness:ti,ab,kw OR 'relational turbulence':ti,ab,kw	123,613
#4	#1 AND #2 AND #3	19,034
#5	#4 AND 'conference abstract'/it	2,788
#6	#4 NOT #5	16,246
#7	#6 AND ([danish]/lim OR [english]/lim OR [norwegian]/lim OR [swedish]/lim)	14,780
#8	#7 AND (2015:py OR 2016:py OR 2017:py OR 2018:py OR 2019:py OR 2020:py OR 2021:py OR 2022:py OR 2023:py OR 2024:py OR 2025:py)	7,802
#9	#8 AND [embase]/lim	843
CINAHL		
#1	(MH "Aged+") OR TI (aged OR old OR older OR elder OR older adults OR senior OR seniors OR octogenarian* OR pensioner* OR dementia*) OR AB (aged OR old OR older OR elder OR older adults OR senior OR seniors OR octogenarian* OR pensioner* OR dementia*)	1,376,792
#2	(MM "Caregivers") OR TI (caregiv* OR "care giv*" OR "care provid*" OR "care staff" OR "community care" OR partner* OR spouse* OR cohabit* OR "co-habit*" OR relative* OR famil* OR sibling* OR sister* OR brother* OR "general practitioner*" OR GP OR GPs OR "occupational therapist*" OR "physiotherapist*" OR "informal carer*" OR couple*) OR AB (caregiv* OR "care giv*" OR "care provid*" OR "care staff" OR "community care" OR partner* OR spouse* OR cohabit* OR "co-habit*" OR relative* OR famil* OR sibling* OR sister* OR brother* OR "general practitioner*" OR GP OR GPs OR "occupational therapist*" OR "physiotherapist*" OR "informal carer*" OR couple*)	1,082,693
#3	((MM "Aging in Place") OR (MM "Home Nursing") OR (MM "Home Health Nursing")) OR TI ("home care" OR "home nursing" OR "care home*" OR "ordinary hous*" OR "ordinary accommodation*" OR "living at home" OR "Ageing in place" OR togetherness OR "relational turbulence") OR AB ("home care" OR "home nursing" OR "care home*" OR "ordinary hous*" OR "ordinary accommodation*" OR "living at home" OR "Ageing in place" OR togetherness OR "relational turbulence")	33,342
#4	#1 AND #2 AND #3	9,362
#5	Publication Date: 20150101-20251231; Language: Danish, English, Norwegian	4,403
PsycInfo		
#1	MM "Older Adulthood" OR TI (aged OR old OR older OR elder OR older adults OR senior OR seniors OR octogenarian* OR pensioner* OR dementia*) OR AB (aged OR old OR older OR elder OR older adults OR senior OR seniors OR octogenarian* OR pensioner* OR dementia*)	719,214
#2	DE "Caregivers" OR TI (caregiv* OR "care giv*" OR "care provid*" OR "care staff" OR "community care" OR partner* OR spouse* OR cohabit* OR "co-habit*" OR relative* OR famil* OR sibling* OR sister* OR brother* OR "general practitioner*" OR GP OR GPs OR "occupational therapist*" OR "physiotherapist*" OR "informal carer*" OR couple*) OR AB (caregiv* OR "care giv*" OR "care provid*" OR "care staff" OR "community care" OR partner* OR spouse* OR cohabit* OR "co-habit*" OR relative* OR famil* OR sibling* OR sister* OR brother* OR "general practitioner*" OR GP OR GPs OR "occupational therapist*" OR "physiotherapist*" OR "informal carer*" OR couple*)	1,194,982
#3	DE "Home Care" OR TI ("home care" OR "home nursing" OR "care home*" OR "ordinary hous*" OR "ordinary accommodation*" OR "living at home" OR "Ageing in place" OR togetherness OR "relational turbulence") OR AB ("home care" OR "home nursing" OR "care home*" OR "ordinary hous*" OR "ordinary accommodation*" OR "living at home" OR "Ageing in place" OR togetherness OR "relational turbulence")	17,014
#4	#1 AND #2 AND #3	5,950
#5	Publication Year: 2015-2025; Language: Swedish, English	2,567
ERIC		
#1	DE "Older Adults" OR TI (aged OR old OR older OR elder OR older adults OR senior OR seniors OR octogenarian* OR pensioner* OR dementia*) OR AB (aged OR old OR older OR elder OR older adults OR senior OR seniors OR octogenarian* OR pensioner* OR dementia*)	112,890
#2	(DE "Caregivers") OR TI (caregiv* OR "care giv*" OR "care provid*" OR "care staff" OR "community care" OR partner* OR spouse* OR cohabit* OR "co-habit*" OR relative* OR famil* OR sibling* OR sister* OR brother* OR "general practitioner*" OR GP OR GPs OR "occupational therapist*" OR "physiotherapist*" OR "informal carer*" OR couple*) OR AB (caregiv* OR "care giv*" OR "care provid*" OR "care staff" OR "community care" OR partner* OR spouse* OR cohabit* OR "co-habit*" OR relative* OR famil* OR sibling* OR sister* OR brother* OR "general practitioner*" OR GP OR GPs OR "occupational therapist*" OR "physiotherapist*" OR "informal carer*" OR couple*)	283,419
#3	TI ("home care" OR "home nursing" OR "care home*" OR "ordinary hous*" OR "ordinary accommodation*" OR "living at home" OR "Ageing in place" OR togetherness OR "relational turbulence") OR AB ("home care" OR "home nursing" OR "care home*" OR "ordinary hous*" OR "ordinary accommodation*" OR "living at home" OR "Ageing in place" OR togetherness OR "relational turbulence")	2,079
#4	#1 AND #2 AND #3	384
#5	Published Date: 20150101-20241231; Language: English	64
SocIndex		
#1	((DE "OLDER people") OR (DE "OLDER men" OR DE "OLDER women")) OR TI (aged OR old OR older OR elder OR older adults OR senior OR seniors OR octogenarian* OR pensioner* OR dementia*) OR AB (aged OR old OR older OR elder OR older adults OR senior OR seniors OR octogenarian* OR pensioner* OR dementia*)	174,008
#2	(DE "CAREGIVERS" OR DE "OLDER caregivers" OR DE "CAREGIVERS -- Social aspects") OR TI (caregiv* OR "care giv*" OR "care provid*" OR "care staff" OR "community care" OR partner* OR spouse* OR cohabit* OR "co-habit*" OR relative* OR famil* OR sibling* OR sister* OR brother* OR "general practitioner*" OR GP OR GPs OR "occupational therapist*" OR "physiotherapist*" OR "informal carer*" OR couple*) OR AB (caregiv* OR "care giv*" OR "care provid*" OR "care staff" OR "community care" OR partner* OR spouse* OR cohabit* OR "co-habit*" OR relative* OR famil* OR sibling* OR sister* OR brother* OR "general practitioner*" OR GP OR GPs OR "occupational therapist*" OR "physiotherapist*" OR "informal carer*" OR couple*)	449,204
#3	DE "HOME care of older people" OR TI ("home care" OR "home nursing" OR "care home*" OR "ordinary hous*" OR "ordinary accommodation*" OR "living at home" OR "Ageing in place" OR togetherness OR "relational turbulence") OR AB ("home care" OR "home nursing" OR "care home*" OR "ordinary hous*" OR "ordinary accommodation*" OR "living at home" OR "Ageing in place" OR togetherness OR "relational turbulence")	6,285
#4	#1 AND #2 AND #3	1,763
#5	Publication Date: 20150101-20251231; Language: Swedish, Danish, English	656

**Figure 1:**
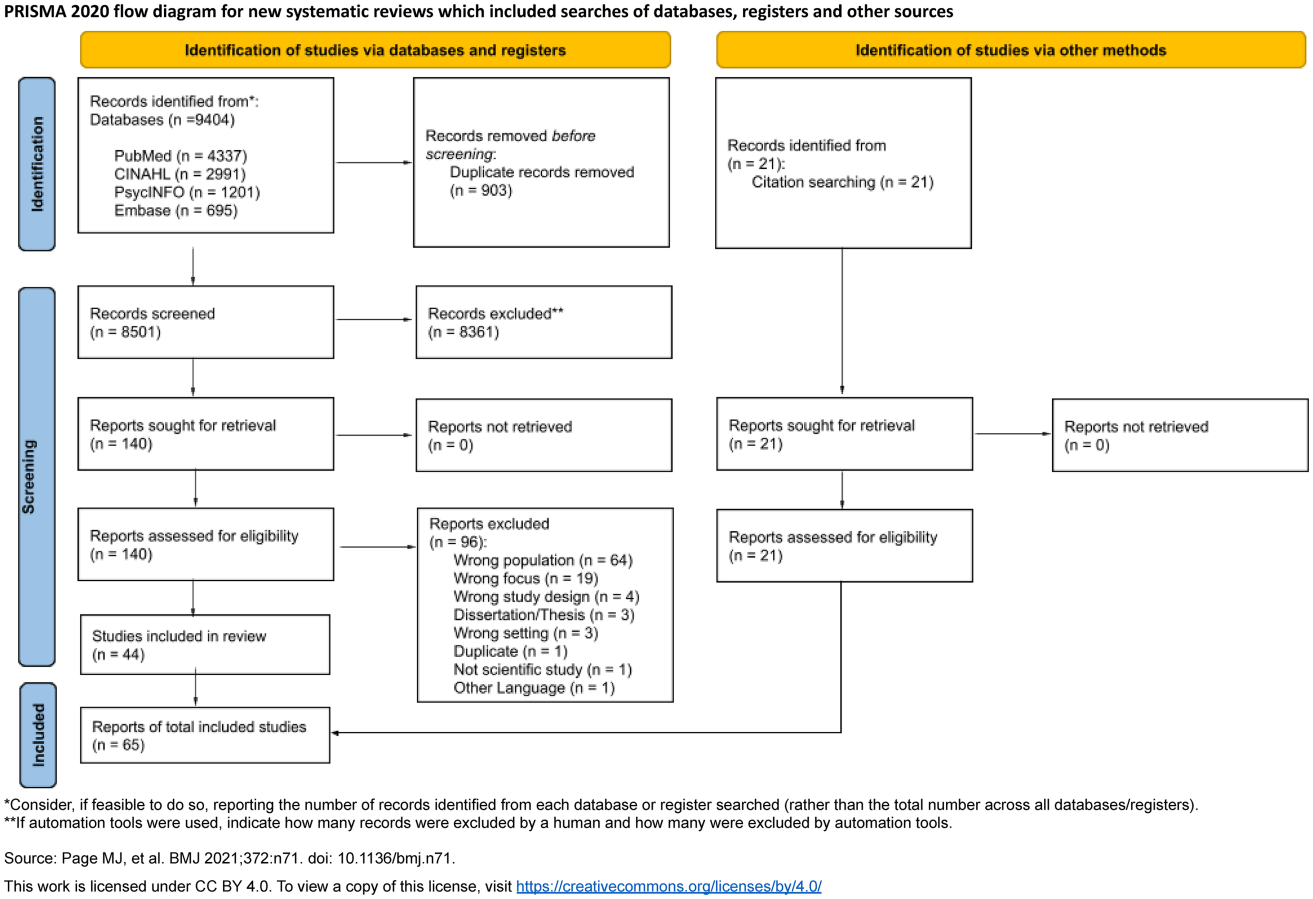
PRISMA 2020 flow diagram for new systematic reviews which included searches of databases, registers and other sources. *Consider, if feasible to do so, reporting the number of records identified from each database or register searched (rather than the total number across all databases/registers). **If automation tools were used, indicate how many records were excluded by a human and how many were excluded by automation tools. Source: Page et al. (41). This work is licensed under CC BY 4.0. To view a copy of this license, visit https://creativecommons.org/licenses/by/4.0/.

The initial search yielded 9,404 publications, which were imported into Covidence software for screening. Two authors (SG and HX) jointly conducted the title and abstract selection process, and three authors (SG, HX and RJAG) the full text screening. To identify additional relevant studies beyond those retrieved using the current search strings, a citation pearl search was conducted in the Web of Science database (Last search: 5 March 2025). This process involved two approaches: (1) examining the reference lists of the included articles to identify further relevant publications, and (2) exploring newer publications that cited the included articles to assess their relevance for inclusion in the current literature review. For any disagreements during screening, full-text review, or pearl search, discussions were held with the co-authors until consensus was achieved. If the two authors (SG and HX) disagreed or were in doubt about a publication’s relevance in the initial screening process, the publication was included in the full-text screening. In cases of disagreement during full-text screening, a third author (RJAG) read the articles and a consensus decision was reached. No disagreements remained regarding final inclusion or exclusion, as discussions mainly concerned articles that addressed the study’s aim only partially in their results. Finally, the third author (RJAG) read and assessed all included publications and supported their inclusion. A PRISMA flow diagram (Figure 1) details the study selection process, with the 65 included publications.

The quality of the included publications was evaluated using the Critical Appraisal Skills Program (CASP) qualitative study checklist (43), ensuring the review’s findings were based on credible, high-quality evidence and reflecting a commitment to methodological rigor. Its use was supported by the Cochrane Qualitative and Implementation Methods Group (44). This checklist comprised 10 questions that assess various aspects of the studies, such as their aims, methodology, design, recruitment strategies, data collection, data analysis, findings, and overall research significance, as detailed in Table 3. The quality appraisal aimed to ensure the robustness of the evidence in addressing our research question.

**Table 3:** Qualitative study appraisal.^*^

Author(s), years	Section A: are the results valid?						Section B: what are the results?			Section C: will the results help locally?	Scores
	1. Was there a clear statement of the aims of the research?	2. Is a qualitative methodology appropriate?	3. Was the research design appropriate to address the aims of the research?	4. Was the recruitment strategy appropriate to the aims of the research?	5. Was the data collected in a way that addressed the research issue?	6. Has the relationship between researcher and participants been adequately considered?	7. Have ethical issues been taken into consideration?	8. Was the data analysis sufficiently rigorous?	9. Is there a clear statement of findings?	10. How valuable is the research?	
Abulaiti et al., 2022 (88)	Yes	Yes	Yes	Yes	Yes	Yes	Yes	Yes	Yes	Yes	10/10
Aaltonen et al., 2021 (74)	Yes	Yes	Yes	Yes	Yes	Yes	Yes	Yes	Yes	Yes	10/10
Andréasson et al. (127) (2023)	Yes	Yes	Yes	Yes	Yes	Yes	Yes	Yes	Yes	Yes	10/10
Atler et al., 2016 (82)	Yes	Yes	Yes	Yes	Yes	Yes	No	Yes	Yes	Yes	9/10
Backhouse et al, 2024 (128)	Yes	Yes	Yes	Yes	Yes	Yes	Yes	Yes	Yes	Yes	10/10
Bendixen et al., 2018 (75)	Yes	Yes	Yes	Yes	Yes	Yes	Yes	Yes	Yes	Yes	10/10
Cao et al., 2022 (83)	Yes	Yes	Yes	Yes	Yes	Yes	yes	Yes	Yes	Yes	10/10
Carabante et al., 2017 (84)	Yes	Yes	Yes	Yes	Yes	No	No	Yes	Yes	Yes	8/10
Cash et al., 2019 (46)	Yes	Yes	Yes	Yes	Yes	Yes	Yes	Yes	Yes	Yes	10/10
Cheng et al., 2024 (92)	Yes	Yes	Yes	Yes	Yes	No	Yes	Yes	Yes	Yes	9/10
Chistell et al., 2023 (89)	Yes	Yes	Yes	Yes	Yes	No	Yes	Yes	Yes	Yes	9/10
Clark et al., 2019 (47)	Yes	Yes	Yes	Yes	Yes	Yes	Yes	Yes	Yes	Yes	10/10
Cole et al., 2022 (56)	Yes	Yes	Yes	Yes	Yes	Yes	Yes	Yes	Yes	Yes	10/10
Compton et al., 2020 (99)	Yes	Yes	Yes	Yes	Yes	Yes	Yes	Yes	Yes	Yes	10/10
Cooper and Pitts, 2022 (80)	Yes	Yes	Yes	Yes	Yes	No	No	Yes	Yes	Yes	8/10
Donnellan et al., 2015 (81)	Yes	Yes	Yes	Yes	Yes	No	Yes	Yes	Yes	Yes	10/10
Greenwood et al., 2019 (100)	Yes	Yes	Yes	Yes	Yes	No	Yes	Yes	Yes	Yes	9/10
Guo et al., 2023 (103)	Yes	Yes	Yes	Yes	Yes	Yes	Yes	Yes	Yes	Yes	10/10
Hale et al., 2020 (57)	yes	yes	yes	yes	yes	No	yes	yes	yes	yes	9/10
Hammar et al., 2021 (48)	yes	Yes	Yes	Yes	Yes	No	No	Yes	Yes	Yes	8/10
Hellström et al., 2017 (58)	Yes	Yes	Yes	Yes	Yes	Yes	Yes	Yes	Yes	Yes	10/10
Hemberg et al., 2018 (129)	Yes	Yes	Yes	Yes	Yes	Yes	Yes	Yes	Yes	Yes	10/10
Hochwald et al. 2022 (96)	Yes	Yes	Yes	Yes	Yes	No	Yes	Yes	Yes	Yes	9/10
Horsfall et al., 2016 (90)	Yes	Yes	Yes	Yes	Yes	Yes	No	Yes	Yes	Yes	9/10
Häikiö et al., 2019 (59)	Yes	Yes	Yes	Yes	Yes	Yes	Yes	Yes	Yes	Yes	10/10
Häikiö et al., 2020 (101)	Yes	Yes	Yes	Yes	Yes	No	Yes	Yes	Yes	Yes	9/10
Jarling et al., 2020 (60)	Yes	Yes	Yes	Yes	Yes	No	Yes	Yes	Yes	Yes	9/10
LaManna et al., 2024 (61)	Yes	Yes	Yes	Yes	Yes	No	Yes	Yes	Yes	Yes	9/10
Larsson et al., 2020 (104)	Yes	Yes	Yes	Yes	Yes	Yes	Yes	Yes	Yes	Yes	10/10
Lethin et al., 2016 (62)	Yes	Yes	Yes	Yes	Yes	Yes	No	Yes	Yes	Yes	9/10
Melilla et al., 2024 (49)	Yes	Yes	Yes	Yes	Yes	Yes	Yes	Yes	Yes	Yes	10/10
Merrick et al., 2016 (50)	Yes	Yes	Yes	Yes	Yes	No	Yes	Yes	Yes	Yes	9/10
Meyer et al., 2016 (63)	Yes	Yes	Yes	Yes	Yes	Yes	Yes	Yes	Yes	Yes	10/10
Miller et al., 2024 (51)	Yes	Yes	Yes	Yes	Yes	Yes	Yes	Yes	Yes	Yes	10/10
Munkejord et al., 2020 (85)	Yes	Yes	Yes	Yes	Yes	No	Yes	Yes	Yes	Yes	9/10
MusgraveTakeda et al., 2022 (64)	yes	yes	yes	yes	yes	yes	yes	yes	yes	yes	10/10
Olivier et al., 2017 (52)	Yes	Yes	Yes	Yes	Yes	No	No	Yes	Yes	Yes	8/10
Papa and Lamura, 2019 (65)	Yes	Yes	Yes	Yes	Yes	Yes	No	Yes	Yes	Yes	9/10
Pedreira et al., 2017 (66)	Yes	Yes	Yes	Yes	Yes	Yes	Yes	Yes	Yes	Yes	10/10
Pejner and Brobeck, 2018 (106)	Yes	Yes	Yes	Yes	Yes	Yes	Yes	Yes	No	Yes	9/10
Pickering et al., 2022 (97)	Yes	Yes	Yes	Yes	Yes	Yes	Yes	Yes	Yes	Yes	10/10
Read et al., 2023 (98)	Yes	Yes	Yes	Yes	Yes	No	Yes	Yes	Yes	Yes	9/10
Redley et al., 2025 (102)	Cannot tell	Yes	Yes	Yes	Yes	No	Yes	Yes	Yes	Yes	8,5/10
Riekkola et al, 2019 (93)	Yes	Yes	Yes	Yes	Yes	Yes	No	Yes	Yes	Yes	9/10
Riekkola et al, 2024 (67)	Yes	Yes	Yes	Yes	Yes	Yes	No	Yes	Yes	Yes	9/10
Rodger et al., 2015 (130)	yes	yes	yes	yes	yes	yes	no	no	yes	yes	8/10
Rykkje and Tranvåg, 2019 (73)	Yes	Yes	Yes	Yes	Yes	Cannot tell	Yes	Yes	Yes	Yes	9,5/10
SadeghiMahalli et al., 2024 (68)	Yes	Yes	Yes	Yes	Yes	Cannot tell	Yes	Yes	Yes	Yes	9,5/10
Schaepe and Ewers, 2018 (105)	Yes	Yes	Yes	Yes	Yes	Cannot tell	Yes	Yes	Yes	Yes	9.5/10
Shiff et al., 2025(53)	Yes	Yes	Yes	Cannot Tell	Cannot Tell	Yes	Yes	Yes	Yes	Yes	9/10
Smith and Shaw, 2017 (78)	Yes	Yes	Yes	Cannot tel	Yes	Cannot tell	Yes	Yes	Yes	Yes	9/10
Stefánsdóttir et al., 2022 (69)	Yes	Yes	Yes	Cannot tel	Yes	Cannot tell	Yes	Yes	Yes	Yes	9/10
Sun et al., 2021 (76)	Yes	Yes	Yes	Yes	Yes	Yes	Yes	Yes	Yes	Yes	10/10
Tatangelo et al., 2018 (94)	Yes	Yes	Yes	Cannot tell	No	Cannot tell	Yes	Yes	Yes	Yes	9/10
Thomas et al., 2018 (86)	Yes	Yes	Yes	Cannot tell	Cannot tell	Cannot tell	Yes	Yes	Yes	Yes	8,5/10
Tolhurst et al., 2023 (70)	Yes	Yes	Yes	Yes	Yes	Cannot tell	Yes	Yes	Yes	Yes	9,5/10
Turjamaa et al., 2020 (87)	Yes	Yes	Yes	Yes	Yes	Yes	Yes	Yes	Yes	Yes	10/10
Turner et al., 2016 (54)	Yes	Yes	Yes	Yes	Yes	No	No	Yes	Yes	Yes	8/10
Tyrrell et al., 2019 (71)	Yes	Yes	Yes	Yes	Yes	Cannot tell	Yes	Yes	Yes	Yes	9,5/10
Vos et al., 2020 (95)	Yes	Yes	Yes	Yes	Yes	Cannot tell	Yes	Yes	Yes	Yes	9.5/10
Wammes et al., 2021 (79)	Yes	Yes	No	No	Yes	Yes	Yes	Yes	Yes	Yes	8/10
White and Palmieri, 2024 (55)	Yes	Yes	Yes	Yes	Yes	Yes	Yes	Yes	Yes	Yes	10/10
Yang et al., 2021 (91)	Yes	Yes	Yes	Yes	Yes	Yes	Yes	Yes	Yes	Yes	10/10
Yang et al., 2023 (77)	Yes	Yes	Yes	Yes	Yes	Yes	Yes	Yes	Yes	Yes	10/10
Zhang et al., 2020 (72)	Yes	Yes	Yes	Yes	Yes	Yes	Yes	Yes	Yes	Yes	10/10

* Conducted in accordance with CASP Qualitative study checklist (43).

### Analytical strategy

The data analysis strategy comprised a descriptive numerical summary analysis, titled ‘Characteristics of the Studies,’ and a reflexive thematic analysis, inspired by Braun and Clarke’s (45) approach. Initially, the publications were read multiple times to ensure thorough familiarisation with the material (45). The following data were extracted by all the authors: (1) Authors, (2) Location, (3) Journal, (4) Study period, (5) Study design, (6) Sample size, (7) Target group and context, (8) Theory/concepts, (9) Results, and (10) Limitations. The focus of data extraction was on the qualitative findings pertinent to the review’s aim and research questions (40). The included studies span a range of contexts and countries, each with distinct cultural and healthcare system characteristics. To manage this diversity, we focused on extracting data relevant across various settings while acknowledging contextual differences. This process was guided by the study’s aim and research questions, and did not require standardized data extraction forms. SG, HX and RJAG verified the extracted data for accuracy and ensured that all relevant results were extracted, and discussed with each other, if they were in doubt. A selection of the extracted data is presented in Table 4.

**Table 4:** Study characteristics.

Author(s), Year of Publication (Country)	Journal (year: impact factor)	Study aim	Design; study population; recruited from; analytical method	Study period
Aaltonen et al., 2021 (74) (Finland)	Dementia (2023: 2.92)	To detect different ways people with memory disorders and spousal carers strive and are able to influence formal care.To recognize situations where their influence on care is described as restricted or even nonexistent.	Semi-structured in-depth life-course interviews (13 dyad/8 individual); 15 older care receiving adults, 19 cohabitant older partners; at home (trough two organisations); Thematic analysis inspired by Braun and Clarke.	October 2018–March 2019
Abulaiti et al., 2022 (88) (China)	Frontiers in Psychiatry (2023: 3.3)	To describe the dyadic care experiences of older adults individuals with disabilities and their caregivers from the perspective of family resilience.	Semi-structured, in-depth interviews; 9 older care receiving adults, 3 cohabitant older partners, 6 other relatives; 4 communities and 2 hospitals; Descriptive phenomenological study, thematic analysis inspired by Colaizzi method and using NVivo 11.0	August 2020–February 2021
Andréasson et al. 2023 (127), (Sweden)	Journal of Family Studies (2023: 1.4)	To explore how the notion of couplehood and family life is understood and negotiated in everyday life by older carers and their spouses.	Ethnographic study with interviews, observations and informal conversations; 7 older care receivers; 9 cohabitant older partners (age: 65+); in and outside couple’s home; abductive, thematic analysis, methodically inspired by Emerson, theoretical informed by Morgan’s sociologically informed theory and conceptualization of family practices and the doing of families/family life.	December 2018–June 2019
Atler et al., 2016 (82) (United States of America)	Physical & Occupational Therapy in Geriatrics (2023: 0.7)	To explore the lived experiences of spousal caregivers providing care to their partners with cognitive changes.	Phenomenological approach by description of the Daily Experiences of Pleasure, Productivity, and Restoration Profile, recording activities over a 24-h period + individual semi-structured interviews + focus group interview; 3 cohabiting older adults (age: 70–83), 2 other relatives; local caregiver support group; thematic analysis	
Backhouse et al, 2024 (128) (United Kingdom)	The Gerontologist (2023: 4.6)	To examine features of personal care interactions between care-home staff and family carers (henceforth collectively termed as caregivers) and people with advanced dementia to understand how care may be improved and inform the development of caregiver educational resources	Naturalistic observation study using one-off video-recorded observations of 26 separate personal care interactions were video recorded hereof 12 interactions from five family caregiver/relative with dementia dyads; 2 cohabitant spouses; 21 other relatives; 16 care-home staff, 42 older care receiving older adults; observational video coding to determine the frequency of actions of people with dementia and qualitative content analysis for in-depth examination	2019
Bendixen et al., 2018 (75) (Norway)	Scandinavian Journal of Caring Sciences (2023: 2.70)	To describe family members’ experiences of attending to an old person with diabetes receiving home care services, including their interaction with the formal caregivers.	Individual semi-structured interviews; 3 co-habitant partners, 5 other relatives; Home care services nurse; Content analysis inspired by Graneheim and Lundman.	May–August 2015
Cao et al., 2022 (83) (China)	Frontiers in public Health (2022: 5.18)	To explore the factors that influence risk perceptions and responses by informal caregivers of older adults with disabilities.	Semi-structured interviews; 5 cohabitant older spouses, 11 other relatives; 6 public organizations having connections with older adults with disabilities; Deductive content analysis based on a socio-ecological framework, using NVIVO	October 2020–February 2021
Carabante et al., 2017 (84) (Sweden)	Scandinavian Journal of Occupational Therapy (2023: 2.74)	To explore and describe how older adults spousal caregivers experience and discuss participation in everyday life when living in shifting contexts due to the use of respite care.	Repeated focus group interviews; 12 cohabitant older partners (10 women, 2 men, age 65–83); A respite care center. Analysis was inspired by a grounded theory approach.	Q1 + Q2, 2014.
Cash et al., 2019 (46) (Australia)	Australasian Journal on Ageing (2019; 1.2)	To explore how expectations of informal care impact spousal caregivers in later life.	Interpretive qualitative design using in-depth interviews; 10 cohabitant older caregivers (age 65–84); Regional Australia. Thematic analysis informed by interpretive qualitative methodology.	Not specified
Cheng et al., 2024 (92) (China)	International Journal of Mental Health Nursing (2024): not published yet. (2023: 5.16)	To elucidate on the experiences of caring and explore the experiences and perceptions of family caregivers in supporting older adults with multimorbidity living in the community to cope with loneliness.	Semi-structured interviews; 3 cohabiting partners, 8 other relatives; a non-government organization (NGO) in Hong Kong, providing community support and home care services; Reflexive thematic analysis inspired by Braun and Clarke.	Not specified
Chistell et al., 2023 (89) (Switzerland)	BMC Nursing (2023: 3.47)	To record and analyse the experience of loneliness among CRs of chronically ill people. Specifically, the aim is to develop a conceptual model based on the concepts of social, emotional, and existential loneliness.	Narrative semi-structured interviews; 10 cohabiting spouses, 3 other relatives; Outpatient care service organizations in Rhaeto-Romanic and German-speaking Switzerland + a regional hospital in Rhaeto-Romanic Switzerland; Thematic analysis, inspired by Saldaña and using MAXQDA software (Analytics Pro 2020).	September 2020–January 2021
Clark et al., 2019 (47) (United Kingdom)	Dementia (2023: 2.92)	To explore the dyadic perspective of dementia within a couple relationship.	Individual semi-structured interviews; 6 cohabiting spouses, 6 partners with dementia; through mental health services for older people within a NHS Foundation Trust; Interpretative phenomenological analysis	Not specified
Cole et al., 2022 (56) (United Kingdom)	Dementia (2022: 2.4)	To investigate the experiences of people living with dementia and their main family carer (family dyad) when managing intimate continence care at home and explore whether this type of care affected their dyad relationship	Semi-structured interviews; 1 older care receiving adult; 7 cohabitant older partners, 6 other relatives; Health and social care organisations, and community organisations supporting people living with dementia; Phenomenological analysis.	Not specified
Compton et al., 2020 (99) (Canada)	Canadian Journal on Aging (2023: 1.7)	To explore the experiences of clients and family caregivers with the services and support provided by Home First, given the complex needs of older adults who want to remain in their home over time.	Semi-structured interviews; 8 older care receiving adults, 8 cohabitant partners, 3 other relatives; ‘First Home’ programme; Thematic analysis inspired by Thorne and Morse.	Not specified
Cooper and Pitts, 2022 (80) (United States of America)	Journal of Social and Personal Relationships (2024: 2.3)	To gain insight into caregiving spouses’ experiences of relational uncertainty and influence from their partner across the prolonged relational transition of Alzheimer’s disease or related dementia (ADRD).	In-depth interviews; 16 cohabitant older partners (9 women, 7 men, age 62–88), 2 other relatives; 3 were widowed; Local memory care center, local Alzheimer’s caregiver support groups, Facebook group; Thematic analysis inspired by Braun and Clarke and relational turbulence theory	January–March 2020
Donnellan et al., 2015 (81) (United Kingdom)	Aging & Mental Health (2014: 2.8)	To assess how spousal dementia carers can achieve resilience and highlight assets and resources they draw on to facilitate or hinder resilience	Individual in-depth semi-structured interviews; 17 cohabitant partners, 2 were widowed and another had their partner admitted in a nursing home; 2 dementia support groups and one care home in North West England; Grounded theory analysis.	Not specified
Greenwood et al., 2019 (100) (United Kingdom)	Maturitas (2019: 3.2)	To explore the experiences of older carers and to understand, from their perspectives, whether their experiences were similar or dierent to those of younger adult carers.	Qualitative study using five focus groups; 44 cohabitant caregivers (age 70–87); Greater London; Thematic analysis.	Not specified
Guo et al., 2023 (103) (China)	BMC Geriatrics (2023: 3.57)	To explore the interaction experience between family caregivers and community nurses for disabled older adults people at home, so as to provide reference signifcance for future related research.	Semi-structured interviews; 2 cohabitant older partners, 5 other relatives, 5 professionals; Linshanzhai Community Health Services Center in Zhengzhou City, Henan Province; Directed content analysis	March–June 2022
Hale et al., 2020 (57) (New Zealand)	The Gerontologist (2020: 5.3)	To report carers’ perceptions of: (a) their role caring for a family member with cognitive decline, (b) the skills and attributes they used to perform this work, and (c) enablers and barriers to achieving their care goals.	Qualitative study using semi-structured interviews; 15 cohabitant caregivers (age 63–89); Community settings in New Zealand; Thematic analysis.	Not specified
Hammar et al., 2021 (48) (Sweden)	Dementia (2023: 2.92)	To explore spouse carers’ experiences of caring for a partner with dementia, their everyday life as a couple and their support needs.	Semi-structured interviews; 9 cohabitant older partners (age 65–94); 2 memory clinics and 2 local support groups of a dementia organisation Latent content analysis inspired by Graneheim and Lundman.	Not specified
Hellström et al., 2017 (58) (Sweden)	Scandinavian Journal of Caring Sciences (2018: 4.6)	To describe how older Swedish men approach the caregiver role of a wife with dementia over time.	Semi-structured interviews; 8 male cohabitant caregivers; Memory clinics; Secondary thematic analysis.	Not specified
Hemberg et al., 2018 (129) (Finland)	Scandinavian Journal of Caring Sciences (2018; 4.6)	To explore and understand experiences of suffering from loneliness in older adults receiving home care.	Hermeneutical inspired individual interviews; 6 cohabitant older adults (aged 72–95), 11 older care recipients living alone; Primary care, Municipality of Ostrobothnia; Latent content analysis inspired by Graneheim and Lundman, informed by a ‘caring science’ theoretical framework.	Not specified
Hochwald et al. 2022 (96) (Israel)	Dementia (2022: 2.5)	To unpack family caregivers’ emotional coping and the emotional-strategies they use; and to place family caregivers’ emotion work within the appropriate Israeli cultural context.	Qualitative phenomenological study using semi-structured interviews; 50 cohabitant caregivers (19 men, 31 women); Home hospice and home care units in Israel; Thematic content analysis.	Not specified
Horsfall et al., 2016 (90) (Australia)	Health and Social Care in the Community (2023: 2.24)	To understand how carers made decisions to accept or reject support as part of the caring journey and to inform policy makers, service managers and providers about how to develop and promote culturally appropriate support services, and negotiate them with carers and care recipients in a timely way	Focus group and individual interviews, standardised tests; 12 cohabitant older partners (age 68–87, the Greek community), 19 healthcare professionals, 6 community leaders; St. George Migrant Resource Centre (SGMRC); Thematic analysis inspired by Braun and Clarke.	2012–2013
Häikiö et al., 2019 (59) (Norway)	BMC Health Services Research (2019: 3.9)	To examine family carers’ perspectives on how to prevent different forms of harm to those living with dementia while receiving community-based services, and how their efforts to alleviate those risks might affect and interact with health professional’s activities in this regard.	Semi-structured qualitative interviews and a consultation of a panel of people with personal or professional experiences; 11 cohabitant older partners, 12 other relatives; A range of health services, institutions or organizations; Thematic analysis inspired by hermeneutic/phenomenological approaches.	June–October 2017
Häikiö et al., 2019 (101) (Norway)	BMC Geriatrics (2020: 4.2)	To explore family carers experiences with, perspectives on, contributions to, and interactions with healthcare services provided to older adults living with dementia.	Qualitative study using semi-structured in-depth interviews; 23 cohabitant caregivers (17 women, 6 men); Healthcare personnel (e.g., dementia coordinators), social media (Facebook), and snowball sampling across Norway (urban and rural areas); Four-step thematic analysis informed by hermeneutic and phenomenological methodology.	June–October 2017
Jarling et al., 2020 (60) (Sweden)	Scandinavian Journal of Caring Sciences (2020: 1.98)	To describe the life situation when family caregivers are imposed responsibility for an older person with complex care needs in their own home.	Individual interviews (a reflective lifeworld research design); 8 cohabitant older partners, 2 other relatives; Primary healthcare; Phenomenological analysis	2017
LaManna et al., 2024 (61) (United States)	Geriatric Nursing (2024: 1.5)	To describe lived experiences of men who engaged in later-life caregiving.	Streubert’s phenomenological qualitative unstructured interview method; 8 older caregivers (age 66–83); Older adult learning communities, caregiver support groups, churches, health fairs, and snowball sampling in Florida, USA; Phenomenological qualitative analysis.	June 2019–January 2020
Larsson et al., 2020 (104) (Sweden)	International Journal of Qualitative Studies on Health and Well-Being (2020: 2.1)	To explore spouses’ existential loneliness when caring for a frail partner later in life	Multi-stage focus group interviews; 5 cohabitant partners, 5 widows; Primary healthcare; Hermeneutical analysis inspired by Dahlberg et al.	August–October 2018
Lethin et al., 2016 (62) (Sweden)	Scandinavian Journal of Caring Sciences (2026: 1.46)	To investigate family caregivers’ experiences of formal care when caring for a person with dementia, through the stages of the disease.	Focus group interviews; 13 cohabitant older partners, 10 other relatives; recruitment via dementia nurses in four municipalities; Content analysis inspired by Graneheim and Lundman and Meleis’ transition theory.	October 2011
Melilla et al., 2024 (49) (Norway)	BMC Health Services Research (2024: 3.9)	To understand the health-promoting experiences of older family caregivers who care for their home-dwelling spouses receiving home-care services	Narrative unstructured interviews; 10 cohabitant older partners (aged 79–91); Primary healthcare; Narrative thematic analysis, inspired by Riessman.	June 2021
Merrick et al., 2016 (50) (United Kingdom)	Dementia (2016: 2.5)	To contribute to our understanding of the experience of dementia from a relational perspective.	interview; Interpretative phenomenological analysis; 7 cohabitant partners (5 men and 2 women); Local branches of the Alzheimer’s Society in the UK; Interpretative phenomenological analysis	Not specified
Meyer et al., 2016 (63) (Sweden)	British Journal of Community Nursing (2016: 0.45)	This study aimed to describe spouses’ experiences of living with a partner affected with dementia	Life-world interviews; 7 cohabitant older partners (4 men, 3 women aged 69–92); recruited via an association for relatives of people affected with dementia; Descriptive phenomenological approach based on a reflective life-world perspective.	Not specified
Miller et al., 2024 (51) (Canada)	Canadian Journal on Aging (2024: 1.5)	To examine husbands whose wives have dementia and how they provide care and construct their sense of self.	Constructivist Grounded Theory using semi-structured interviews; 11 older caregivers (age 61–88); Caregiver and memory support organisations, clinics, social media, and snowball sampling in Ontario, Canada; Constant comparative analysis.	May–June 2021
Munkejord et al., 2020 (85) (Iceland and Norway)	International Practice Development Journal (2020: 1.79)	To provide a deeper understanding of the struggles, suffering and unmet needs of care partners by listening to the voices of older women living with and caring for a spouse with severe cognitive decline.	In-depth open-ended interview; 11 older partners (some still cohabiting, some widowed); Primary care (Norway), nursing home (Iceland), private persons through advertisement in a newspaper Norway; Thematic analysis.	2018–2019
Musgrave-Takeda et al., 2022 (64) (Japan)	Dementia (2022: 2.4)	To identify the experience of being the spouse of a person with dementia in the context of their marital relationship	Observation and semi-structured interviews; 7 cohabitant older partners (4 male and 3 female); recruited from managers at home nursing facilities; hermeneutic Heideggerian phenomenological analysis.	Not specified
Olivier et al., 2017 (52) (New Zealand)	Scandinavian Journal of Caring Sciences (2017: 0.37)	To explore the lived experience of three stroke family members during the 18 months following a first-ever stroke.	Stand-alone case study; individual conversational style interviews at 6 weeks, 12 months and 18 months; 1 cohabitant older partner, 2 other relatives; Hospital; Thematic phenomenological analysis following van Manen	September 2011-September 2013
Papa, and Lamura, 2019 (65) (Italy)	Journal of Gerontology and Geriatrics 2019 (2019: 0.27)	To provide evidence of informal caregivers pivotal role in care provision	Semi-structured face-to-face in-depth interviews; 2 cohabitant older partners (age 72 and 80), 4 other relatives; the Italian National Institute of Health and Science on Ageing; Framework Analysis	November–December 2017
Pedreira et al., 2017 (66) (Brazil)	Journal of Clinical Nursing (2017: 3.2)	To understand the lived experience of older Brazilian carers.	Semi-structured interviews; 3 cohabitant older partners (73–84 years old), 3 other relatives (63–78 years old); public home-care programme in Salvador (north-eastern Brazil); hermeneutic phenomenological analysis.	January–February 2016
Pejner and Brobeck, 2018 (106) (Sweden)	Home Health Care Management and Practice (2018: 0.8)	To describe how couples in need of home care services experienced the received support from care professionals	Focus groups; 8 cohabitant older partners, 8 older care receiving persons (couples) aged between 65–80 years old, 2 nurses; Relative Association (nonprofit for family caregivers) and home care of the municipality; content analysis.	Not specified
Pickering et al., 2022 (97) (Canada)	Health & Social Care in the Community (2022: 2.5)	To explore the transnational systems of support that Canadian spousal caregivers use to provide care while living seasonally in the United States as international retirement migrants.	in-depth semi-structured dyad interviews; 20 cohabitant partners (age > 60); Facebook groups for Canadians in Yuma and postcards on Canadian-plated cars; Thematic analysis.	January 2019
Read et al., 2023 (98) (United Kingdom)	Parkinson’s Disease (2023:2.1)	To facilitate an in-depth exploration and further comprehend the lived experience of caregiving for late-stage Parkinson’s and the perception of service needs and provision from the family-caregivers’ perspective in England.	Semi-structured interviews; 6 cohabitant older partners, 5 other relatives; the English cohort of the European “Care of Late-Stage Parkinsonism” (CLaSP) study with help from general practitioners’ (GPs) surgeries, NHS hospital outpatient clinics, Parkinson’s charities, and specialist neurologists in and within Greater London; Thematic analysis inspired by Braun and Clarke.	2016
Redley et al., 2025 (102) (United Kingdom)	Healthcare (2023: 1.95)	Explore how family caregiver experience input from a team managing crises in dementia (TMCD)	Semi-structured interviews; 4 cohabitant older partners, 3 other relatives; Primary health care; Thematic analysis	Not specified
Riekkola et al, 2019 (93) (Sweden)	Journal of Aging Studies (2019: 1.54)	to explore how older adults couples, who are in need of social services in the community, act and reason over time regarding their everyday togetherness	Shared interviews and participant observations; 3 female co-habitant spouses and their 3 male ill partners (age 66–78); Recruited by a caregiver counselor at one municipality; Data analysis followed Polkinghorne’s description of the paradigmatic analysis of diachronic narrative data.	November 2016–February 2018
Riekkola et al, 2024 (67) (Sweden)	Journal of Aging Studies (2024: 2.24)	To explore and describe the experiences and reasoning of spousal carers, healthcare professionals, and stakeholders regarding possibilities for older couples to age in place.	Focus groups; 12 cohabitant older partners (age 65–83, 10 women, 2 men), 18 healthcare professionals, 16 stakeholders; Residential respite care facility in a municipality; Constant comparative methodology inspired by Charmaz	Not specified
Rodger et al., 2015 (130) (Ireland)	British Journal of Community Nursing (2015: 0.4)	To explore the experiences of informal carers in Ireland and to identify supports required in caring for older adults at home	Unstructured interviews; 1 cohabitant older partner (80 years old), 5 siblings or children; outpatient clinic in an older person service in Ireland; Morse and Field’s 4-step Heideggerian hermeneutic phenomenological analysis.	2009
Rykkje, and Tranvåg, 2019 (73) (Norway)	SAGE Open (2023: 2.0)	To explore the experiences of husbands engaged in caregiving for their home-dwelling spouse with dementia.	Qualitative individual interviews; 5 cohabitant husbands (age 72–82 years); 2 hospital memory clinics; Exploratory design founded upon Gadamer’s philosophical hermeneutics, a four-step hermeneutical analysis	Not specified
Sadeghi-Mahalli et al., 2024 (68) (Iran)	Geriatric Nursing (2023: 2.5)	To explore the support process for older spousal caregivers of people with Alzheimer’s disease.	Semi-structured, in-depth interviews; 10 cohabitant older partners, 3 other relatives, 3 healthcare providers; One memory clinic and one care center; Grounded theory analysis inspired by Corbin and Strauss’s method, using Word software.	2022–2023
Schaepe and Ewers, 2018 (105) (Germany)	BMC Nursing (2023: 3.1)	The study aims to explore family caregivers in Home Mechanical Ventilation (HMV) safety experiences and how safety is perceived by them in this context; it seeks to understand how family caregivers contribute to the patients’ and their own safety in HMV and what kind of support they expect from their health care team.	Exploratory semi-structured interviews; 6 cohabitant older partners, 3 other relatives; Nursing care providers, respiratory care center, a health care insurance company, personal contacts and organizations; Thematic analysis, inspired by Braun and Clark among others, using the software MAXQDA 11	June 2014–June 2015
Shiff et al., 2025 (53) (United States of America)	Journal of Applied Gerontology (2023: 2.2)	We sought to explore dementia caregiving experiences from the perspective of spouses/partners; identify common motivating factors and greatest challenges associated with how and why spouses/ partners provide in-home care for their loved one living with dementia.	Mixed methods longitudinal from 2 studies (validated tools, interviews, observations); 15 older care receiving adults, 15 cohabitant partners (aged 65–90); recruitment site not specified in San Francisco, CA; Secondary data analysis using Thematic Analysis inspired by Braun and Clarke.	Study 1: 2018–2020 Study 2: 2021–2024
Smith and Shaw, 2017 (78) (United Kingdom)	Medicine, Health Care and Philosophy (2023: 2.3)	To explore family members’ lived experience of Parkinson’s disease and their opportunities for well-being.	In-depth interviews; 4 older care receiving adults, 5 older cohabitant partners; One Parkinson’s support group; Interpretative phenomenological analysis.	Not specified
Stefánsdóttir et al., (69) (Iceland and Norway)	Scandinavian Journal of Caring Sciences (2023: 1.9)	To shed light on couplehood changes as experienced by men caring for wives with dementia.	Individual in-depth interviews; 8 cohabitant husbands (67–92 years); Primary care; Constructivist grounded theory study, data analysis inspired by Charmaz, using NVivo software	2018–2019
Sun et al., 2021 (76) (Canada)	Geratric Nursing (2023: 2.5)	To gain a better understanding of the relationship between client’s therapeutic self-care ability and homecare safety outcomes, and the role of self-care and caregiving activities in supporting homecare safety in relation to chronic disease management.	One-on-one, in-depth, semi-structured interviews; 15 older care receiving adults 65 + and 15 cohabitant older partners (15 dyads); One homecare organization in Ontario; Qualitative description/naturalistic inquiry and thematic analysis inspired by Patton.	Not specified
Tatangelo et al., 2018 (94) (Australia)	International Journal of Nursing Studies (2023: 7.5)	To examine the health needs of partner and offspring caregivers of older people with dementia, including the barriers they experience in meeting their needs.	Semi-structured interviews; 12 cohabitant older partners (aged 62–89), 12 other relatives; [Setting unknown]; Thematic analysis approach, using NVivo software.	Not specified
Thomas et al., 2018 (86) (United Kingdom)	Palliative Medicine (2023: 3.6)	To illustrate the relevance of ‘relevant background worries’ in family carers’ accounts of caring at home for a dying adult	Qualitative cross-sectional observational (in-depth semi-structured interviews); 30 caregivers; General practitioner (GP) practices; Narrative analysis presented as 4 case studies (3 cohabitant older partners and 1 daughter)	2011–2012
Tolhurst et al., 2023 (70) (UK)	Healthcare (2023: 2.5)	To explore how couples negotiate relationships and care following a dementia diagnosis, with a focus on the perspectives of male caregivers.	Semi-structured interviews; 10 female older care receivers adults, 10 male cohabitant partners (aged 62–86); 2 dementia support groups and one church organisation; A thematic analysis founded upon a constructivist and interpretivist framework.	Not specified
Turjamaa et al., 2020 (87) (Finland)	Healthcare (2020: 1.6)	To describe the individual experiences of older caregivers who were looking after a spouse with a memory disorder	Thematic individual interviews; 10 older co-habitant partners (6 women, 4 men, age 69–86); One memory clinic at a health center; Inductive content analysis	During spring 2016
Turner et al., 2016 (54) (United Kingdom)	Age and Ageing (2023: 6.0)	To explore the experiences of the ‘oldest carers’ in caring for a dying spouse at home.	In-depth interviews; 17 cohabitant older partners (aged 80–90); Primary care in the North West (Lancashire and Cumbria) and South West (East Devon) of England; Thematic analysis inspired by Braun and Clarke, Ritchie and Spencer, and Reissmann.	2011–2013
Tyrrell et al., 2019 (71) (Sweden)	Dementia (2023: 2.4)	To describe spouses’ experiences of living with partners who have developed neuropsychiatric symptoms related to dementia in a community setting.	Semi-structured interviews; 14 cohabitant older partners (aged 64–85); Older adult clinics, one older adult psychiatry unit, one dementia support organisation; Content analysis inspired by Krippendorff.	November 2014–November 2015
Vos et al., 2020 (95) (The Netherlands)	Health and Social Care in the Community (2023: 2)	This study aims to explore older adults’ experiences of changes in their social networks and to understand the impact of these changes on their lives.	Focus Groups; 14 cohabitant older partners (aged 65+); Four home-care organisations; Grounded Theory analysis.	April–May 2017
Wammes et al., 2021(79) (Netherlands)	Alzheimer’s and Dementia (2021: 4.9)	To prioritize care characteristics for community-dwelling persons with dementia and informal caregivers using innovative-mixed-methods approach	Focus groups with a quantitative ranking exercice; 10 cohabitant carers, 7 children, 2 relatives and 1 close friend; 5 day-centers across the Netherlands and a dementia-support organization; thematic analysis through Braun and Clarke’s approach.	December 2019 to March 2020
White and Palmieri, 2024 (55) (United States of America)	International Journal of Qualitative Studies on Health and Wellbeing (2024: 2.6)	To describe the lived experience of women caregivers of male spouses living at home with Parkinson’s disease	Semi-structured interviews; 12 female cohabitant carers aged between 60 and 83; recruited from the Colorado Parkinson Foundation; phenomenological analysis using Colaizzi’s seven-step process.	Not specified
Yang et al., 2021 (91) (China)	International Journal of Nursing Practice (2023: 1.9)	This study aimed to explore the experiences of family caregivers interacting with people with dementia.	Descriptive phenomenological qualitative inquiry using semi-structured interviews; 5 cohabiting older partners, 5 other relatives; Department of Neurology and Mental Health in Hangzhou in Zhejiang Province; Thematic analysis inspired by Braun and Clarke, using NVivo9	June–September 2018
Yang et al., 2023 (77) (Taiwan)	The Journal of Nursing Research (2023: 2.4)	The aim of this study was to explore the care experiences of FCs caring for older family members with cancer at home.	In-depth interviews; 5 cohabitant older partners, 17 other relatives; Chemotherapy outpatient setting of a medical center in northern Taiwan; Content analysis inspired by Graneheim and Lundmann.	January–December 2019
Zhang et al., 2020 (72) (China)	Dementia (2023: 2.4)	This study aims to explore the meaning of family supported home care in China from the perspectives of people with dementia and family caregivers.	In-depth, semi-structured individual interviews; 10 care receiving older adults, 5 cohabitant older partners, 9 other relatives; Shandong Mental Health Centre; Thematic analysis inspired by Braun and Clarke.	August 2016–January 2017

**Figure 2:**
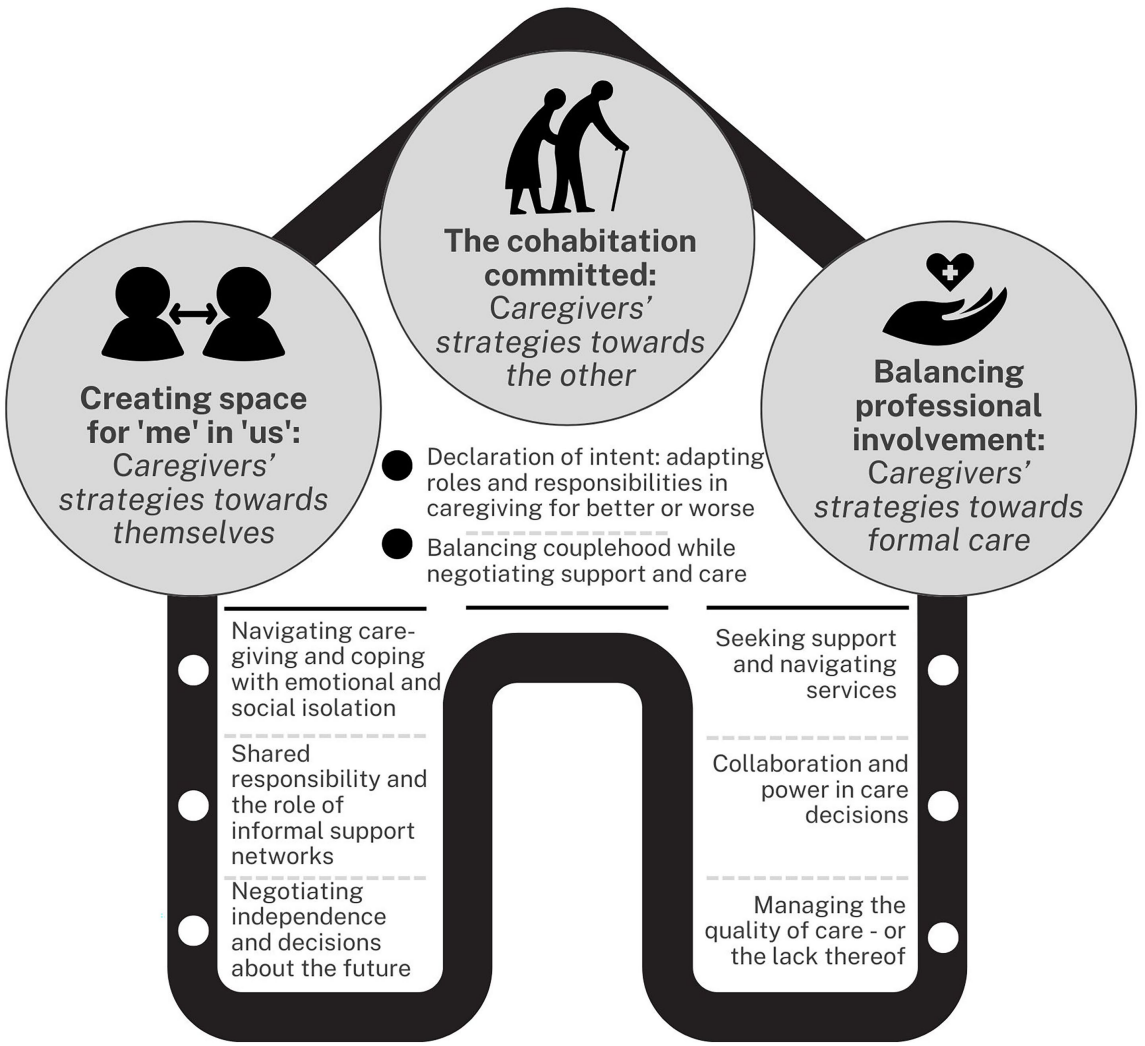
Themes and subthemes.

The result sections of the publications were initially coded and then reorganised to align with the review’s research questions (45). From these codes, preliminary themes were developed by examining patterns of similarity and difference. Codes with similar meanings were clustered together to form overarching themes. The themes were reviewed and refined through a collaborative process among the authors. This involved multiple iterations, where the themes were revisited alongside the empirical data and research questions to ensure that the themes accurately reflected the data (45). In the final stage, each main theme and its sub-themes were clearly defined, refined, and named. They were thoroughly reviewed to ensure they were both succinct and sufficiently descriptive (45). The resulting themes and subthemes are presented in Figure 2.

## Results

### Characteristics of the studies

The studies were conducted in Sweden (*n* = 12), United Kingdom (*n* = 12), Norway (*n* = 7), China (*n* = 6), United States of America (*n* = 5), Canada (*n* = 4), Australia (*n* = 3), Finland (*n* = 3), Iceland (*n* = 2), Netherlands (*n* = 2), New Zealand (*n* = 2), Brazil (*n* = 1), Iran (*n* = 1), Ireland (*n* = 1), Israel (*n* = 1), Italy (*n* = 1), Japan (*n* = 1), Germany (*n* = 1), Switzerland (*n* = 1), and Taiwan (*n* = 1). Almost all studies were conducted in one country, and two studies were conducted in two countries. A total of 1,103 participants took part in the included studies, distributed as 682 cohabiting caregiver partners, 164 older care receiving adults, 175 other relatives, 66 healthcare professionals and 16 stakeholders. All studies used qualitative methods, where most studies (*n* = 50) used individual interviews, eight studies used focus group interviews, and one study used video recordings. Six studies used both observations and interviews. Ten different analytical methods were used where 26 studies conducted thematic analysis, 12 used content analysis, 11 applied a phenomenological analysis, five used a grounded theory analysis, four applied a hermeneutic phenomenological analysis, two used a hermeneutic analysis, and five used other analytical methods, see Table 4.

The older care receivers were described as living with dementia (*n* = 31), chronic diseases (*n* = 14), disabilities (*n* = 6), and other mixed medical diagnosis (*n* = 14). Twenty-seven studies focused primarily on older partner caregivers. Ten studies included both the cohabiting caregiver and the care receiver. Nineteen studies included other family members as well as the older partner caregiver and/or the care receivers. Other eight studies also included healthcare personnel. The articles were published in journals with a specific focus on health(care) science (*n* = 17), dementia (*n* = 10), gerontology/geriatrics (*n* = 15), nursing (*n* = 8), psychiatry or mental health (*n* = 3), and others (*n* = 11). The impact factor of the journals ranged from 0.27 to 7.5, see Table 4.

According to the authors’ assessment using the CASP checklist (43), all selected publications demonstrated appropriate methodological rigour (Table 3). Overall, CASP scores ranged between 8/10 and 10/10, and the majority of included studies had scores of 9 or higher (56 studies out of 65), suggesting excellent quality. One common area of limitation was the relationship between researchers and participants (Criterion 6), either unclear or unreported in 28 out of 65 studies, followed by the recruitment strategy (Criterion 4), insufficiently described in six studies (Table 3).

### The cohabitation committed—caregivers’ strategies towards the other

#### Declaration of intent—adapting roles and responsibilities in caregiving for better or worse

Several caregivers described a commitment to fulfil the moral obligation of the partnership whilst adopting a strategy to manage their emotions and feelings, and work on them and change them in alignment with certain ideals and perceived marital expectations (46–55, 127). They revealed shifts in household and care responsibilities following illness or disability, with many cohabiting caregivers commonly experiencing the need to adapt to new roles and expanded duties (46–51, 53, 55–72, 127).

Several cohabiting caregivers found evident shifts in domestic tasks, with caregivers taking over tasks previously managed by their partners, including cooking, grocery shopping, home maintenance, and financial responsibilities (46–51, 53, 55–70, 72, 127). For many male cohabiting caregivers, the traditional gendered divisions of household was altered and they had to learn and perform activities traditionally associated with female roles, however their strategies drew upon using skills as leadership and problem-solving, gained through prior life challenges and work experiences (46, 49–51, 56, 58, 61, 62, 73). In addition to household tasks, many caregivers transitioned into more professional roles, including medication management, monitoring health conditions, assistance with mobility and hygiene, and interactions with health and social care systems (46–51, 53, 55–72, 74–77, 127).

Some caregivers used Information and Communication Technology (ICT) for medical documentation, health tracking apps to help train the memory, and care coordination, acting as experts without necessary support from the health care system (127). Other caregivers described the need for close follow-up to remind their partners to use their assistive technologies such as portable alarms, GPS-tracking, phones and other safety measures (59). Some cohabiting caregivers developed strategies of being present and able to adapt to shifting needs and tailor their support, finding assistive technologies helpful, as they promoted independence (59, 78, 79). However, some caregivers reported that these roles were often accompanied by a lack of familiarity and formal guidance and thus led to uncertainty and confusion (56, 59, 62, 64, 76, 80).

Many caregivers experienced emotional and identity challenges as they adjusted to expanded responsibilities (46–51, 53, 55–65, 67–72, 127). With the progression of illness, caregivers’ responsibilities grew while their personal freedom diminished. Some caregivers linked their current situation to serving a prison sentence (65, 81), or a loss of freedom (60). Cohabiting caregivers and care receivers emphasised the need for constant presence and supervision as key strategies to prevent harm, such as falls or accidents (54, 59, 60, 70, 82–87). However, such responsibility with ongoing presence and supervision often led to emotional exhaustion and a sense of being overburdened among caregivers, especially when care receivers resisted help in an effort to preserve independence (59, 60, 82, 84, 85, 88).

#### Balancing couplehood while negotiating support and care

Many cohabiting caregivers expressed that caregiving shifted the dynamics of couplehood, which led to their relationships characterised by mutual vulnerability, where both partners beared physical and emotional burdens and uncertainty in face of illness and caregiving (46, 48, 50, 55, 59, 60, 62, 64, 70, 73, 81, 82, 89, 90, 127). They experienced tensions between their roles as being a partner and a caregiver, describing a strategy of taking a more pragmatic and task-oriented approach to the situation and the practicalities that needed to be done (47, 56, 58, 60, 74, 127). Many highlighted the tension between respecting their partner’s independence and the increasing need to intervene for safety and well-being (55, 59, 70, 82, 127).

Caregivers often engaged in emotional work to preserve their partners’ dignity and identity, actively striving to maintain the marital relationship instead of letting illness and dependency completely redefine it (46–48, 50, 53, 55, 57–59, 64, 69, 70, 127). There were examples of caregivers that might balance the act of being a lover and being a caregiver, reminiscing the important and good times, and receiving small verbal and non-verbal gestures seemed to be a coping strategy for the change in the attentiveness of their partner (47, 49, 53, 64, 80, 89). A common strategy mentioned was keeping a positive climate between the partners, avoiding triggering anger or hostility, often by putting their own needs second (48, 57, 59, 91). Doing so seemed to get easier when understanding the disease and its different symptoms and how it affected the person they cared for (59, 70, 86). However, the overwhelming responsibilities sometimes caused caregivers to put their lives on hold to focus on caring for the other, at the expense of their own social and emotional needs (49, 66, 89).

A strategy used by some cohabiting caregivers was to redefine responsibility, viewing their new roles and tasks as a privilege that brought purpose to their changed circumstances and to life more broadly (54, 56, 87, 90). Despite noticeable changes in reciprocity between partners, some caregivers adopted the strategy of focusing on the positive aspects of the relationship during caregiving as a way to cope and support their own well-being (50, 71). However, there seemed to be a diversity in the need for maintaining an active life outside of caregiving, providing pleasure, productivity and restoration while others chose to use their time with the care receiver (69, 82).

### Creating space for ‘me’ in ‘us’—caregivers’ strategies towards themselves

#### Navigating caregiving and coping with emotional and social isolation

Caregivers often paused their own lives to dedicate themselves to caring for the other, at the expense of taking care of their own illnesses and physical ailments (52, 54, 65, 66, 79, 83–86, 90, 92–95) and their own social and emotional needs (49, 66, 89). Loneliness was closely tied to caregivers’ internalised sense of responsibility, often leading to extended isolation and hopelessness, as well as feelings of frustration, resentment, and guilt directed at the care receiving partner (64, 65, 82, 87, 89).

Caregivers employed myriad strategies to reduce loneliness and preserve normalcy, emphasising self-care, meaningful activities, and relationships beyond their caregiving role to support their wellbeing (48, 49, 73, 84, 87, 89, 91, 95). Often this included socialising, engaging in hobbies and activities, and using technology to stay connected. In some cases, pets offered relief from loneliness (89). Many caregivers sought social contact outside of their relationship with friends and family (55, 57, 61, 84, 87, 90, 91, 127), such as caregiver support groups to bond over shared experiences (48, 79, 82). Others carved out time for skill-based classes, outdoor physical activities or leisure/hobbies, or to read or watch television programs on their own (49, 55, 64, 77, 82, 84, 87, 89). Some caregivers found relief in their faith and spirituality (55, 77). Some caregivers even opted to include their partner during activities rather than miss out on the opportunity to participate (73, 78) or found time for themselves by maintaining activities in their partners’ lives by keeping them engaged in enjoyable pastimes despite their declining condition (81).

Use of social technology was also a strategy utilised by caregivers to both get a break from caregiving activities and to socially and emotionally connect with others. It was a way to find new online friendships (127). In some cases, when care recipients spent time on the computer, caregivers could also take a break from caregiving and run errands (127). Social media sites and apps (e.g., Facebook, Instagram) provide opportunities to connect with people in similar caregiving situations (48) and stay in touch with friends and family (93, 127). Some caregivers noted that video calling apps (i.e., Zoom) seemed too impersonal of a platform for sharing their situation (55). Technology such as smartphones and tablets offered a variety of distracting app-based games, some of which had an embedded social element played virtually with others. A shared interest of, e.g., watching boats and the use of a boat information app provided entertainment and connection to beloved pastimes aided by technology during in-person interactions (127).

However, some caregivers adopted emotional distancing or passive endurance as a strategy, or because of a result of a lack of external support (48, 58, 88, 89, 96). In some cases, caregivers became apathetic, stating there was ‘nothing they could do’ or they were ‘fed up’ (88). Loneliness even manifested as a physical pain for some caregivers (89). The overwhelming responsibilities associated with caregiving had some partners wishing for death on themselves (48) or their partners to relieve the suffering of both partners (48, 96). Yet at the same time, caregivers also expressed fear of the loneliness they will feel when their partner dies, which was deemed worse than the burden of caregiving itself (96). Few caregivers opted for divorce (58, 96).

#### Shared responsibility and the role of informal support networks

Many caregivers adopted a strategy of actively seeking support from family, friends and neighbours to manage their daily lives as caregivers (49, 54, 55, 60, 62, 68, 83, 84, 87, 90, 93, 97, 98). Such support consisted of assisting with household maintenance (98), preparing of food (97), helping with the physical and emotional care of the ill partner (55, 85), emotional support of the cohabiting caregiver (7, 49, 97), and enabling outdoor activities for the cohabiting caregiver (84). The support also consisted of advice and information provision (65, 83, 98). Some caregivers benefitted from having a close family member being a health professional (57), or a retired health worker as neighbours who could address medical complications (97). Others were struggling to find the right information and support (65). The amount of support received from family and friends differed greatly. Cohabiting caregivers recognised factors such as travel distance (84), family obligations, and health issues limited the support they received from family and friends (54). Support from others could ease the cohabiting caregiver’s burden by creating a sense of shared responsibility (60). However, some received little support and had to beg for help (67), while others feared becoming a burden (84). Other cohabiting caregivers first assessed a person’s ability and willingness to provide effective support before asking for help (68). Some caregivers also viewed faith communities as part of their informal support network (98).

Connecting with caregiver support groups was another strategy to manage daily life (48, 55, 59, 68, 70, 79, 81, 82, 87, 97). Caregiver support groups offered help through the sharing of experiences (55, 68, 70, 79, 81, 97), benefitting from knowing other people who were experiencing similar situations (55), easing feelings of guilt and helping to normalise their feelings (48, 59), sharing information (68, 70, 79, 81, 97). Several caregivers were happy to be able to share the information they had compiled (55, 68), giving them a feeling of becoming experts (81). They were more likely to use informal support networks if they could offer social support to others in the same situation encouraging independence and “giving back” rather than dependence (81). However, support groups did not necessarily suit everybody and were described as helpful to a certain point (48). Some cohabiting caregivers got anxious when the amount of information was impossible to digest (87). Other caregivers received support from other people in similar circumstances on the internet if they were not able to leave the house (48). Others again did not have experiences with caregiver support groups but asked for the opportunity to join one (85).

#### Negotiating Independence and decisions about the future

The utilization of respite care constituted a deliberate strategy by caregivers to manage their caregiving responsibilities, safeguard their own well-being, and facilitate the continued residence of the care recipient in the home environment for as long as feasible (48, 58, 63, 67, 84, 87, 93). Respite care enabled caregivers to complete tasks such as grocery shopping (48) and gardening (58), regaining their energy (93), emotional and physical recovery, engaging in meaningful activities, and maintaining essential social relationships (67). It also offered time to grieve, reflect on life’s changes, and consider future living arrangements (84). Others hesitated to use respite care because they were reluctant to send their partner away from home (87), or feared the emotional consequences of doing so (84, 93).

While the use of respite care represented one aspect of the caregiving strategy, the decision to transition the care recipient to a nursing home was a considerably more consequential and emotionally fraught choice (56, 63, 68, 85, 89, 90, 99), especially when the care recipient refused (85, 89, 99), or when cohabiting caregivers believed that care homes offered inferior care or could even pose a fatal risk to their relative (56). Cohabiting caregivers frequently encountered significant uncertainty regarding the trajectory of the care recipient’s condition and the implications for their own caregiving role (47, 55, 62, 63, 65, 78, 91). This uncertainty often centered on concerns about disease progression (65, 78), the caregiver’s capacity to maintain their responsibilities over time (62, 65, 94), and the anticipated disruption of shared daily life with their partner (47, 63). In response, caregivers employed various strategies to manage the psychological and practical demands of their role. Some engaged in proactive planning, while others adopted a present-focused approach, deliberately avoiding long-term considerations as a means of emotional self-regulation (91). For certain individuals, the recognition of inevitable decline led to a strategic emphasis on the present moment, reflecting a perceived lack of control over future outcomes (80).

### Balancing professional involvement—caregivers’ strategies towards formal care

#### Seeking support and navigating services

Seeking and navigating care services were central and often challenging aspects of life for caregivers in a cohabiting couple. As a result of shifting responsibilities, many caregivers had to be more proactive in identifying and accessing formal support, with home care services playing a key role in sustaining ageing at home (46, 47, 54, 56, 58, 59, 62, 67, 70, 74, 79, 84, 90, 92, 99–102). Other caregivers demonstrated a reluctance to accept public services, reflecting personal preferences and societal expectations of independence (46, 73, 74). Some caregivers found formal care easily accessible (46, 99, 101), but this was not a straightforward process. Some caregivers had to fight for services, switch to new, lower-quality products (e.g., incontinence aids), navigate opaque bureaucratic systems, manage financial matters like reimbursements, and deal with shifting regulations (56, 62, 67, 74, 79, 99–101). Access to formal care was not taken for granted by caregivers, knowing that overuse could affect others’ access or reflect negatively on their caregiving abilities (99). Cultural and linguistic accessibility was also viewed by some caregivers as key to comfort and communication through care navigation (90). In some cases, caregivers described using social leverage, through other family members or appealing to higher authorities, to obtain needed care (101).

While caregivers frequently performed many tasks themselves, formal services like home care support helped with essential activities of daily living, such as dressing, medication adherence or practical challenges, like incontinence (e.g., (49, 54, 69, 79)), which enabled them to attend to their other needs or responsibilities (79, 99). Beyond home care support, caregivers collaborated with other health professionals, including physical and occupational therapists, district nurses, general practitioners, and palliative care nurses (54, 92, 99). Some caregivers also recognised the importance of professional input for their own well-being, expressing a desire for greater involvement of doctors or social workers in addressing issues like loneliness and fatigue (92) and rationalising their life situation (47, 62). In other cases, caregivers were left entirely unsupported, having to beg for help or deal with inexplicable service withdrawals (67). Other caregivers sought alternative treatments through personal research and online purchases when conventional support felt limited (70). While some caregivers found creative ways to engage with services, others struggled with barriers that left them feeling isolated and overwhelmed.

#### Collaboration and power in care decisions

When balancing their responsibilities, several caregivers asserted their desire to be involved in decision-making (103) and to maintain control over the delivery of care and the cohabiting couples’ daily living (56) while avoiding intrusion in their intimacy and routines (84). However, they often faced challenges in establishing trustful relationships and collaboration with formal care providers (49, 56, 62, 66, 74, 84, 85, 99, 103). Some caregivers described feeling disempowered when formal caregivers dismissed their input and observations (49, 55, 69, 74, 87, 100, 103). Uncertainty about receiving support at a specific time disrupted some caregivers’ daily routines, often leaving those caregivers to resort to carrying out the tasks themselves (84) or to conclude that it would have been simpler without (74). Often, caregivers recognised that most attention by formal care was around the older care recipients, where the needs and responsibilities of caregivers themselves were not recognised or considered; for example, the need for their own daily schedule to be synchronised with the delivery of home care services and caregivers’ well-being (48, 49, 55, 66, 69, 87, 103). When responsibilities became overwhelming (49) or when disease-related complications occurred (85, 103), caregivers recognised the value of formal care (49, 62, 74, 89, 99, 100, 103), especially when the scope of tasks was overwhelming (49) or when they lacked knowledge about disease-related complications (85, 103).

To avoid repeating past suboptimal care experiences (59), some caregivers strove for more control over the care provision as a result of mistrust (59, 61, 74). The presence of formal caregivers might also be experienced as an intrusion in the home, which became a contested space, both physically (e.g., bedrooms) and relationally (56) and re-allocating of rooms (e.g., bedrooms) (86). Some caregivers found this intrusion difficult, to the point of limiting or rejecting formal support, even when needs were extensive (84). In some cases, involvement of formal carers was perceived as a threat to their caregiving role (90), leading to feelings of judgment or exclusion or ‘not fitting in’ (48, 56). In other cases, caregivers viewed the company of the health professionals as a way to reduce their potential loneliness (89, 90). The continuous dialogue with health professionals was essential to adapt strategies when previous ones failed (59, 84). Other caregivers feared being displaced by professionals or other family members, signalling an assertion of their authority within the dyad (74, 104). Struggling between being decision-makers and the ones left behind, caregivers often faced morally conflicting decisions (74), where revealing symptoms, such as aggressive behaviours (48), could unintentionally mean exposing their partner to harm or institutionalisation (104).

From the perspective of some caregivers, building trust with ‘allied’ staff members became a vital strategy for gaining influence, others also relied on private communications with professionals to convince them of their views on the care situation (74). This happened when professionals included family carers as part of the team (99, 105). Many caregivers found that a strong relationship with formal care professionals, marked by mutual recognition of roles, compassionate communication, and information sharing, could improve service responsiveness (62, 103), particularly in urgent situations (99). Such relationships were seen as key to the success of own caregiving strategies (57, 62, 63, 75, 98, 100, 103, 105).

#### Managing the quality of care—or the lack thereof

Successful collaboration with formal care providers was associated with quality of care when professionals demonstrated competence, continuity and accommodation to the reality of caregivers, offering problem-solving support and social interaction (48, 49, 57, 62, 63, 67, 75, 82, 98, 99). Such collaboration was highly dependent on the invisible labour of informal caregivers, involving advocacy efforts, coordination, and monitoring (49, 55, 74, 86, 101, 105). Some caregivers preferred not to make all decisions (104) and took a more hands-off role (81), mainly monitoring daily care (59).

This invisible labour was applied specifically to fill systemic gaps and failures resulting from under-resourcing and fragmented services (68, 86, 99, 101). Caregivers often described needing to advocate persistently or persuade professionals to adapt care to their lived reality (74). Some caregivers filled in where formal services failed, for example, by providing hands-on care in hospitals (66), preparing for emergencies (105), and coaching professionals (105). In some cases, the trust in professionals was so low that caregivers preemptively trained staff to ensure safe care delivery (105) or preferred doing the care themselves (48, 66). Frequent changes in staff (86) and limited number of home visits (67) as well as inadequate transfer of information (e.g., discharge notes) created burdens for some caregivers who had to ensure care continuity (48, 101). Other resources like respite care were perceived as inconsistent with caregivers’ desire to maintain normalcy and avoid stigmatisation, e.g., being labelled as part of another generational group (57). The act of ‘surrendering’ a partner to others’ care was experienced as both necessary and devastating, fraught with doubt, grief, and the fear of abandonment (104).

Sometimes, caregivers strategically withheld information to steer decisions (74), revealing underlying tensions between the perceived expertise of professionals and caregivers’ intimate knowledge (106). Some caregivers weighed the risk of deteriorating relationships with service providers against the potential benefits of advocating for better care (101). Strategic efforts were deployed to maintain person-centredness in a context of heavy care standardisation (71, 106) and professionalisation (56), which often was translated into levelling down care quality by ignoring individual needs and emotional aspects (48, 71, 85, 101). As such, managing care quality was not just about ensuring appropriate medical treatment; it was an active and often moral engagement with a system that frequently fell short.

## Discussion

The discussion focuses on two main findings, namely a role transition from being a partner to being a caregiver and the new responsibilities and strategies associated with it, and how the strategy of involving formal care at home combined practical, moral, and emotional labour of the older cohabiting caregivers to ensure good care for their partner. Furthermore, the method’s strengths and limitations are discussed.

The findings revealed a role transition from being a cohabiting partner to becoming both a partner and a primary caregiver that brought new responsibilities and strategies with it. According to van Gennep (107), a transition process often begins with a phase of separation. This involves a gradual detachment from the familiar, shared identity as equal partners. Typically triggered by the onset of illness, frailty, or disability, the caregiving partner becomes increasingly aware that their relationship is no longer defined solely by mutuality but is now shaped by new roles, responsibilities and dependencies. This stage may be marked by emotional turmoil, including a sense of loss and anticipatory grief, as the couple’s previous balance begins to dissolve and the structures that supported their shared life begin to shift (108, 109). Following separation, van Gennep (107) describes a liminal phase in the transition, which is defined as an ambiguous space characterised by uncertainty and ambivalence. In this state, the individual is no longer simply a partner, but not yet fully identified with the caregiving role, capturing the in-betweenness of this experience. The findings showed how the caregiving partner inhabits a dual role, navigating the emotional demands of intimacy and companionship alongside the practical and moral demands and responsibilities of care. This role is often unstable and fraught with internal tensions. At the same time, they are engaged in an ongoing moral negotiation about what they owe their partner, how much they can realistically give, and how their own needs and well-being fit into the equation. Liminality is not only experienced individually, but can also affect the couple’s shared identity, as they renegotiate what it means to be together in a context of increasing asymmetry (110). Over time, the caregiving partner may reach a stage of incorporation (107), wherein they re-enter the social world with a newly stabilised identity with the development of strategies that work as well as possible for the balance of the partnership and for the individual caregiver partner. At this point, they may begin to self-identify as a caregiver and receive external recognition in that role. This incorporation was, as shown in the findings, reinforced through strategies of social activities, participation in support groups, engagement with health and social services, or adjustments in daily life and routines. However, incorporation is not always a neat or complete process. The nature of long-term caregiving means that roles continue to evolve, and the balance between care and companionship often remains in flux. Even so, as the findings revealed, many caregivers found strategies to integrate elements of their former partnership, such as shared rituals, emotional closeness, or mutual recognition, into this new phase of life, resulting in a layered and complex sense of identity. The shift from partner to caregiver is not merely practical but is a significant social and moral transformation (111). It involves crossing multiple thresholds in terms of identity, relationship, and social status, including changed responsibilities within partnership and the development of new everyday strategies. As the findings pointed out, the transition towards being a caregiver was also a question about the health conditions of the cohabiting caregiver. In many older cohabiting couples, both partners are ill or frail (112, 113). However, the neoliberal governance of care support at home often assumes that the less ill partner can take responsibility and act on behalf of the couple (114). Yet unlike traditional rites of passage, this transition is often unmarked by formal rituals or societal recognition, making it an invisible yet deeply consequential process in the lives of ageing couples, including responsibility and role distribution. Traditional gendered roles typically push women to take on caregiving roles more often than men, and those caring for older adults are less likely to be paid for their labour, which paradoxically restricts their availability for paid work (115). The overlapping roles of caregiving and couplehood leaves little room for self-care, leisure, and even paid work, thereby reinforcing social isolation, marginalisation and undermining health and economic security (116). As it often occurs in private households, informal caregiving remains hidden from policy frameworks or formal systems of support, uncaptured through conventional institutional measurements (117). To better support caregivers in these transitions to older couplehood and ageing-in-place, future studies should explore the rites of passage in older cohabiting couples when life conditions change and new roles and responsibilities emerge.

Furthermore, the findings showed different strategies in which informal caregivers actively engaged with formal care systems to maintain their life at home. Caregivers often walk a thin line between self-reliance and institutional dependency, continually reassessing what is ‘enough,’ ‘acceptable,’ and ‘possible’ in the shifting landscapes of care. As shown in the findings, caregivers frequently described navigating services as a continuous and often burdensome responsibility and strategy. Funk (118) shows that navigation supports remain fragmented and condition-specific, leaving many older adults and caregivers struggling to access care. At political and provider levels, this calls for patient-centred strategies, including improving information, expanding public support, and integrating services (118). The support of home care services, particularly for tasks such as personal hygiene, mobility, and medication management, was acknowledged as essential to sustaining ageing-in-place. However, access to these services was rarely straightforward or reliable. Instead, caregivers were required to become strategic agents, continuously evaluating, combining, and supplementing formal resources to meet complex and evolving care needs. This suggests a form of practical-moral reasoning, wherein carers make judgments not only about what is possible, but about what is right and necessary in their particular circumstances (119). Seeking support is not merely a logistical task; it is an ongoing moral practice shaped by care ethics, social inequalities, emotional strain, and systemic (dis)function, as also shown by Lilleheie et al. (120). For some, accepting help from outside the family represents a failure of moral responsibility or a breach of relational commitment. For others, particularly those navigating progressive care needs, it is a necessary, even urgent, adaptation to protect both themselves and their partners. Care systems require caregivers to become ‘moral entrepreneurs’ (121), who must advocate, argue, and even battle for access to support (122). The findings highlighted that cohabiting caregivers expressed frustration with inconsistent or absent follow-up from services, necessitating a proactive stance just to obtain basic help. This reveals a troubling dynamic. Even when caregivers formally ‘belong’ to the care system, they are expected to demonstrate their worthiness or urgency through persistence, suggesting a system that implicitly delegates responsibility onto the very people it is meant to support (123, 124). At the same time, caregivers’ efforts to navigate and coordinate services also reveal forms of agency, creativity, and resilience. The results suggested that some found ways to integrate different supports, combining formal rehabilitation with respite care or learning techniques from professionals to better manage behavioural symptoms. These practices can be understood as situated acts of moral repair (119) in which caregivers attempt to restore a sense of order and coherence amid fragmented care environments. However, the emotional toll of this work is significant. The burden of constant form-filling, follow-up calls, and struggles for consistency was not only exhausting but demoralising, as shown in the findings. Caregivers sometimes felt ignored or unheard, and when services were subpar, it was experienced not just as a failure of quality, but as a violation of the personhood and dignity of the person cared for. Such failures represent a breach in the moral fabric of care, undermining the trust and mutuality that caregivers strive to uphold. Older cohabiting caregivers operate within care systems that are at once enabling and limiting, requiring them to negotiate ethical tensions between duty, exhaustion, and systemic inadequacy (119). The unevenness of support thus reflects not only structural fragmentation but also a failure to recognise the moral significance of caregivers’ knowledge, efforts, and experiences. This pointed to the need for more responsive, relationally attuned services that do not just provide care, but actively support the moral labour of caregiving itself. These findings call for Ageing-in-place policies that explicitly recognise the invisible, morally-driven labour undertaken by cohabiting caregivers. To ensure the relevance and responsiveness of national and local eldercare strategies, caregivers’ perspectives must be considered in the design and evaluation of care programmes. In addition, it is important that policymakers address issues such as broken agreements and inadequate support by implementing stronger quality assurance mechanisms and accessible complaint procedures, also bridging formal and informal care systems. Future studies about the moral labour of caregiving are needed to understand the contextual and relational complexities in the encounters between informal and formal caregiving when ageing-in-place.

### The study’s strengths and limitations

The study has several strengths and limitations. For pragmatic reasons, the results were based on articles that included cohabiting partners aged 60 and above. However, the notion that age can be reduced to a mere number oversimplifies the complexity of human experience, biological diversity, and the social influences that shape the ageing process. Firstly, biological ageing is not uniform. Individuals of the same chronological age can differ significantly in physical health, cognitive function, and overall vitality. Secondly, the social construction of age imposes rigid expectations. Society assigns roles, privileges, and limitations based on chronological age, from birth to death. Yet, these categories are often arbitrary and fail to reflect individual capabilities. Moreover, emotional and psychological ageing do not always align with numerical age depending on the lived lives. Defining a person’s stage of life solely by the number of years lived disregards these nuances. While using age as a numerical measure may be convenient, it is an inadequate and overly simplistic representation of the ageing process (30). As with most literature reviews, it was not possible to cover the full range of conditions experienced in relation to caregiving, with nearly half of our studies focusing on dementia (31 out of 65 studies). Whereas dementia brings complex relational challenges greatly affecting the caregiving experience, this overrepresentation might also be the result of a sampling limitation, where more demanding conditions are more frequently reported than other, less demanding ones. Our qualitative interpretation, combined with the detailed context of each study, supports the transferability of our findings, thus addressing this limitation, and future studies focusing on a broader range of experiences are encouraged.

While our results touched upon the gendered aspects of caregiving, a more explicit focus could have illuminated critical nuances in this review and enriched our interpretations, and these should be addressed in future studies. The review covers studies published between 2015 and 2024, spanning pre- and post-COVID-19 contexts. Although the pandemic likely intensified challenges such as isolation, reduced service access, and increased moral labour, these dynamics were unevenly addressed across studies. Future research could more directly examine how caregiving roles shifted during and after the pandemic. The included studies also span 20 countries with diverse healthcare systems, welfare models, and cultural understandings of family care. Such heterogeneity shapes how caregiving is supported and experienced, for instance, strong formal care infrastructures may ease family responsibilities, whereas family-based systems place greater demands on relatives (125). Cultural norms around gender, ageing, and obligation further influence how moral labour is perceived and enacted. While this diversity enriches the current review, it limits direct transferability of findings. Future research should investigate how systemic and cultural factors mediate caregiving experiences to inform context-sensitive policy and practice. Furthermore, only relevant articles in English were found. It appears that the formal search did not lead to articles in French or Scandinavian languages; even the Pearl Search did not locate such articles. Yet, articles do exist; for example, Vedsegaard and Wind (112). However, many lower-ranked journals are not indexed in the major scholarly databases. In addition, English has become the leading language in academia, the lingua franca (126), which is why most research is published in English-language journals. We used the Web of Science database to ensure the inclusion of newer publications that cited these articles, assessing their relevance for the current literature review. It seems that the initial search was not precise enough, as about a third of the included articles were found through pearl search. One explanation is that articles that did not use the term ‘older adults’ to describe this group of people have been difficult to capture in the search. For example, Aaltonen et al. (74) did not use the term ‘old*’ or ‘older adults’ but used the term ‘people with memory disorders.’ A subsequent review of the articles found through pearl search also reveals that other articles could have been found by adding keywords such as ‘family living’ and/or ‘couplehood’ to the primary searches, e.g., Andréasson et al. (127). However, the first author subsequently discussed the uncaptured articles with the expert university librarian involved, concluding that the extensive pearl search had successfully identified the articles missed in the initial search. Furthermore, the search found a lot of articles related to the aim, but many of the articles did not separate older adults from other caregivers in the results. All articles where it was not possible to distinguish cohabiting older adults (caregivers) from other caregivers were excluded. This means that the results clearly represent this group, but at the same time, additional knowledge about this group may be present in the excluded studies, which could not be differentiated in this literature review.

## Conclusion

Focusing on the perspectives of cohabiting older caregivers, the results showed that when transitioning from their roles as partners to that of caregivers, cohabiting older adults transformed the couple’s relationship to enact new responsibilities. Caregivers took responsibility for both their partner and for holding together fragile systems of care. Their work was driven not just by necessity, but by a commitment to sustaining relationships, honouring personhood, and doing what they understood to be ‘the right thing,’ even when systems failed to adequately support them. This pointed to the need for more responsive, relationally attuned services that do not just provide care, but actively support the moral labour of caregiving itself. This also calls for user-involving research and participatory designs within home care, with the aim of supporting the needs of older adult cohabiting couples in a time when political trends advocate for ageing-in-place.

## Data Availability

The original contributions presented in the study are included in the article/supplementary material, further inquiries can be directed to the corresponding author.
