# Correction: Older caregivers' responsibilities and strategies for their cohabiting partners living at home—a qualitative systematic literature review

**DOI:** 10.3389/fpubh.2025.1734743

**Published:** 2025-11-10

**Authors:** Stinne Glasdam, Hongxuan Xu, Ruth-Ellen Slåtsveen, Christie Stilwell, Gitte Wind, Ragnhild Julante Andersen Gulestø, Pier-Luc Turcotte

**Affiliations:** 1 Department of Health Sciences, Lund University, Lund, Sweden; 2 Department of Care Science, Malmo Universitet, Malmö, Sweden; 3 Centre for Development of Institutional and Home Care Services in Oslo Kommune, Oslo, Norway; 4 Faculty of Health, Dalhousie University, Halifax, NS, Canada; 5 Department of Nursing and Nutrition, Kobenhavns Professionshojskole, Copenhagen, Denmark; 6 Department of Health Sciences, VID Vitenskapelige Hogskole, Oslo, Norway; 7 University of Ottawa, Ottawa, ON, Canada

**Keywords:** ageing-in-place, caregiver burden, cohabiting partners, older adults, qualitative systematic literature review, responsibilities, roles

The **Acknowledgments** section was mistakenly omitted. It appears below:

“The authors thank Krister Aronsson (Librarian at support for research and learning, Library & ICT, Faculty of Medicine, Lund University) for his dedicated assistance and work in designing and performing the literature searches.”

The original version of this article has been updated.

